# Knee Loading Asymmetries During Descent and Ascent Phases of Squatting After ACL Reconstruction

**DOI:** 10.3390/app15147780

**Published:** 2025-07-11

**Authors:** Manuel Angel Romero Padron, Alyx Jorgensen, David M. Werner, Matthew Alan Tao, Elizabeth Wellsandt

**Affiliations:** 1 Department of Orthopaedic Surgery and Rehabilitation, University of Nebraska Medical Center, 4014 Leavenworth St., Omaha, NE 68105, USA; 2 Physical Therapy Program, University of Nebraska Medical Center, 4014 Leavenworth St., Omaha, NE 68105, USA; 3 College of Allied Health Professions, University of Nebraska Medical Center, 3908 Jones St., Omaha, NE 68198, USA

**Keywords:** rehabilitation, biomechanics, movement asymmetry, ACL, neuromuscular control

## Abstract

Asymmetries are common during squats following anterior cruciate ligament reconstruction (ACLR). This study examined interlimb loading differences between squat phases at 6 months post-ACLR. Thirty-five participants performed bodyweight squats at self-selected speed and were analyzed using 3D motion capture. Vertical ground reaction force impulse (vGRFi), external knee flexion moment impulse (KFMi) and hip-to-knee flexion moment impulse ratio (HKRi) were calculated, along with interlimb ratios (ILR). Squat phase durations were also recorded. Paired t-tests and ANCOVA (controlling for time) were used to compare biomechanical variables across squat phases. Greater asymmetry was observed during ascent for vGRFi ILR (*p* = 0.045), KFMi ILR (*p* < 0.001) and HKRi ILR (*p* = 0.006). The ascent phase was faster than descent (*p* = 0.036). After adjusting for time, phase-related differences in ILRs were no longer significant. These findings suggest that greater limb and knee-specific loading asymmetries occur during the ascent phase of squats but may be influenced by movement speed. Importantly, significant knee-specific loading asymmetries persisted regardless of squat phase. At 6 months post-ACLR, addressing neuromuscular control and movement speed during rehabilitation may help reduce biomechanical imbalances during closed kinetic chain exercises.

## Featured Application

This study shows that squat phase and speed affect interlimb loading asymmetries after ACL reconstruction. Clinicians should monitor movement tempo and target neuromuscular control during rehabilitation to reduce asymmetries and improve outcomes during closed kinetic chain exercises.

## 1. Introduction

Squats are a commonly performed exercise during rehabilitation after anterior cruciate ligament (ACL) reconstruction (ACLR) [1,2]. During early- and mid-rehabilitation stages after ACLR, intralimb compensations during squats that shift loading toward the hip and ankle joints of the injured limb, as well as shifted loading to the non-injured limb, occur [3,4]. These compensatory movement patterns often persist after patients have completed rehabilitation, as seen up to 7 years after ACLR [5]. Restoring symmetric movement patterns is important because altered knee joint loading is associated with negative outcomes such as a second ACL injury and the development of knee osteoarthritis (OA) [4,6-8].

Previous research has focused on loading asymmetries during the entire length of squats (descent combined with ascent phase) in individuals after ACLR. A study performed by Sigward et al. compared the injured vs. uninjured limb of individuals after ACLR during the entire length of squats, finding that individuals exhibited lower peak knee flexion angles, knee extension moments and peak vertical ground reaction forces while squatting at 3 and 5 months after surgery [1]. Through investigations of squat biomechanics by Sanford et al. and Garrison et al., the injured knee of individuals after ACLR was compared to healthy controls during the entire length of squats, with increases in anterior femoral translation relative to the tibia, asymmetric ground reaction forces, increased hip energy absorption contributions and decreased knee energy absorption contributions present [5,9]. While previous studies have demonstrated squat-related asymmetries, the distinct phases of the squat [descent and ascent] impose different demands on the musculoskeletal and neuromuscular systems (e.g., knee extensors elongate during descent and shorten during ascent), warranting phase-specific investigation [10,11]. Only the work performed by Strong et al. described squat asymmetries during the descent and ascent phases of squats [4]. However, their data compared the injured knee after ACLR to a single limb of healthy controls without measures of interlimb asymmetries, reporting greater differences in knee flexion and ankle flexion moments during both the descent and ascent phases at 2.9 months and the descent phase alone at 8.8 months post-ACLR (*p* < 0.05) [4]. Nonetheless, the comparison of interlimb squat biomechanics during the descent compared to the ascent phase of squats remains absent from the current literature.

The purpose of this study was to determine if loading symmetries differ between the descent and ascent phases of bilateral squats at 6 months after ACLR and if the length of time during each phase influences squat loading patterns. We hypothesized that interlimb loading asymmetries would be present and similar during the descent and ascent phases of the bodyweight squats. Findings from this study may inform more phase-specific assessment and rehabilitation strategies aimed at reducing persistent movement asymmetries and optimizing knee joint loading after ACLR. Ultimately, reducing asymmetries during functional movements may help decrease the risk of early osteoarthritis and reinjury following primary ACL reconstruction.

## 2. Materials and Methods

### 2.1. Study Design

This is a cross-sectional secondary analysis from a prospective cohort study aiming to determine the association between joint loading and cartilage degeneration after ACLR [12]. Participants were enrolled pre-operatively early after sustaining an ACL injury and followed through 18 months after ACLR. The timepoint of interest in this study was 6 months after ACLR.

### 2.2. Participants

Participants between the ages of 15 and 35 who were within one month of ACL injury at the time of enrollment were eligible for the larger prospective study [12]. Exclusion criteria included previous knee injury or surgery in either knee, history of inflammatory disease, immune compromise, chronic use of NSAIDS, history of cortisone injection during prior three months, current pregnancy, contraindications to MRI, contraindications to MRI, symptomatic concomitant grade III tear to other knee ligaments requiring surgical intervention, a pre-operatively planned meniscectomy by the treating orthopaedic surgeon and acute or degenerative chondral lesions present on post-injury MRI. All participants provided written informed consent (minors provided assent with written consent from a parent or legal guardian).

#### Participant Characteristics

Participants self-reported age, sex and race. All study data were collected and/or managed using Research Electronic Data Capture (REDCap) electronic data capture tools hosted at UNMC. REDCap is a secure, web-based application designed to support data capture for research studies [13,14]. REDCap at UNMC is supported by the Research IT Office, funded by the Vice Chancellor for Research (VCR). Operative reports were collected from each participant’s surgeon and were used to identify graft type and meniscus repair.

### 2.3. Squats

Three-dimensional motion analysis was used to examine participants’ bilateral squats. Kinematic and kinetic data were simultaneously collected with two embedded force platforms (1080 Hz; Bertec Corporation, Columbus, OH, USA) and an eight-camera motion capture system (120 Hz; Qualisys Inc, Goteborg, Sweden). Passive retroreflective markers (14 mm) were placed on anatomical landmarks of the trunk, pelvis and lower extremities as previously described, including the sternum, C7, T10 and bilateral acromia, upper back, posterior superior iliac spine (PSIS), iliac crest, anterior superior iliac spine (ASIS), medial and lateral femoral epicondyles, medial and lateral malleoli, superior and inferior heel and the first and fifth metatarsal heads [15]. In addition, rigid shells, each containing four retroreflective markers, were secured bilaterally at the lateral thigh and shank.

Participants first completed a 1-s static trial in anatomical position [15]. After this calibration trial, the markers on the ASIS, femoral epicondyles, malleoli and first metatarsal heads were removed prior to dynamic trials. As part of a larger testing protocol (gait, single-leg squats, drop vertical jumps), participants then completed three sets of five bilateral squats with arms crossed over the chest [16]. They were instructed to squat to a comfortable depth at a self-selected speed without further instruction or practice.

Marker trajectories were labeled in Qualisys Track Manager and exported to Visual3D (HAS-Motion, Kingston, ON, Canada). Target and ground reaction force (GRF) data were low-pass filtered using a fourth-order bidirectional Butterworth filter with a 12 Hz cutoff frequency [1].

A subject-specific model including the trunk and lower extremities was created using measured height and mass to estimate segment lengths and joint centers as previously described [1]. To account for the marker diameter and its base, virtual landmarks were offset by 9 mm toward the underlying bone [17]. Virtual ASIS and PSIS landmarks were used to create a Visual 3D composed pelvis. Hip joint centers were estimated using the method of Bell et al., knee joint centers were defined as the midpoints between the virtual femoral epicondyle landmarks, and ankle joint centers were defined as the midpoints between the virtual malleolar landmarks [18,19].

**Figure 1.**
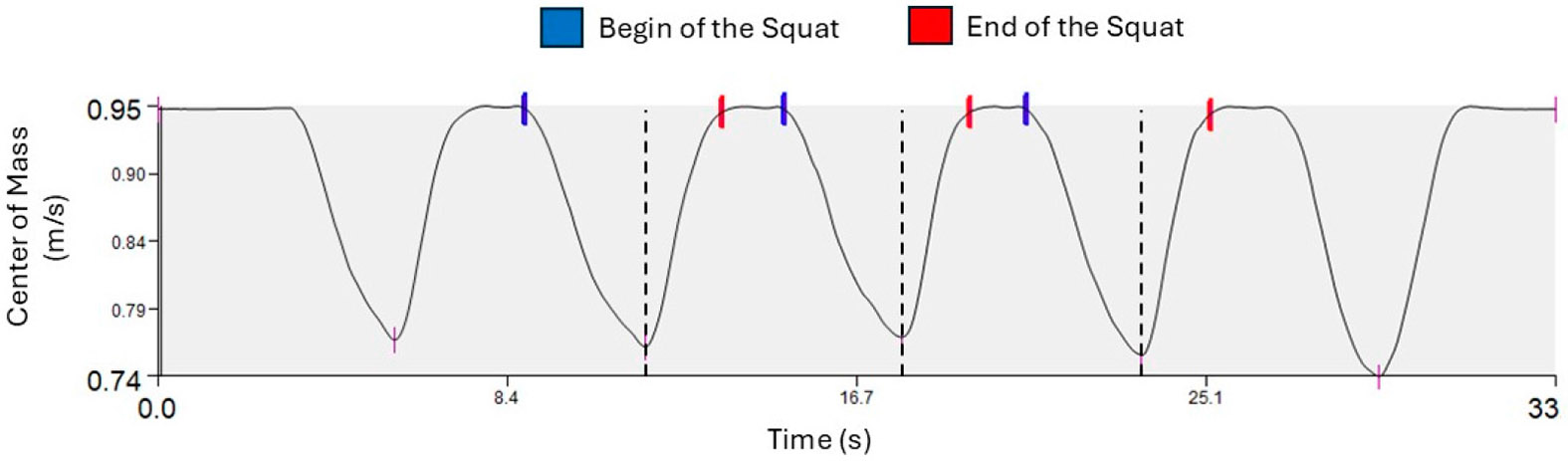
Graphical representation of the body’s center of mass (COM) in the vertical direction. The vertical velocity of the COM was used to define the beginning and end of each squat. The middle three of each set of five squats were used for analysis. Dashed lines represent the end of the descent phase and the beginning of the ascent phase, defined by the lowest vertical position of the COM. Abbreviations: m/s, meter per second; s. seconds.

Each squat was segmented into descent and ascent phases. The start of the squat was defined when vertical center-of-mass velocity exceeded 0.02 m/s downward, and the end of the squat was defined when upward center-of-mass velocity decreased below 0.02 m/s (Figure 1). The lowest vertical center-of-mass position determined the transition point between the descent and ascent phases. External knee and hip moments were calculated using an inverse dynamics approach, and joint impulses for the descent and ascent phases were computed using the trapezoidal rule [20].

Variables of interest included vertical ground reaction force impulse (vGRFi), external knee flexion moment impulse (KFMi), ratio of external hip flexion to knee flexion moment impulse (hip-to-knee ratio (HKRi)) and time spent in the descent and ascent phase. Data from the 3 middle squats (second, third and fourth) of each set of squats (9 total squats averaged) was averaged to account for more variable movement patterns during the first and fifth squats of each set [1]. For each variable, an interlimb ratio (ILR) was calculated using this equation: involved limb/uninvolved limb. An ILR of 1 indicates an equal distribution of load between limbs (vGRFi), between knees (KFMi) or between the hip and knee joints across limbs (HKRi). An ILR of less than 1 for vGRFi and KFMi indicates lower loading in the injured limb or knee compared to the uninjured limb or knee. An ILR greater than 1 for the HKRi indicates a greater contribution of loading in the hip than the knee in the injured limb compared to the uninjured limb. Time spent during the descent and ascent phases was expressed in seconds.

### 2.4. Statistical Analysis

Statistical analyses were completed using IBM SPSS Statistics (IBM Corp. Released 2022. IBM SPSS Statistics for Windows, Version 30.0. IBM Corp: Armonk, NY, USA). Demographic and surgical characteristics were described using means and standard deviations or frequencies and percentages, as appropriate. First, paired t-tests were performed to compare each biomechanical variable (vGRFi, KFMi, HKRi) between phases. One outlier for HKRi was detected that was more than 1.5 box-lengths from the edge of the box in a boxplot. It represented expected variation within the sample population and thus was kept in the analysis. Three extreme outliers for HKRi were detected that were more than 3.0 box-lengths from the edge of the box in a boxplot. These 3 outliers were removed from the analysis of HKRi due to their leverage on statistical results. Assumptions of normality were not violated, as assessed by visual inspection of histograms and P-P plots. Secondly, an ANCOVA was used to determine if ILRs for each squat variable differed between phases after controlling for time. There was a linear relationship between time on each phase and each of the biomechanical variables for each of the phases, as assessed by visual inspection of a scatterplot. Standardized residuals were normally distributed, as assessed by Shapiro–Wilk’s test (*p* > 0.05). There was homoscedasticity and homogeneity of variances, as assessed by visual inspection of a scatterplot and Levene’s test of homogeneity of variance, respectively. There were no outliers in the data, as assessed by no cases with standardized residuals greater than ±3 standard deviations. A *p*-value of 0.05 was set a priori.

## 3. Results

**Figure 2.**
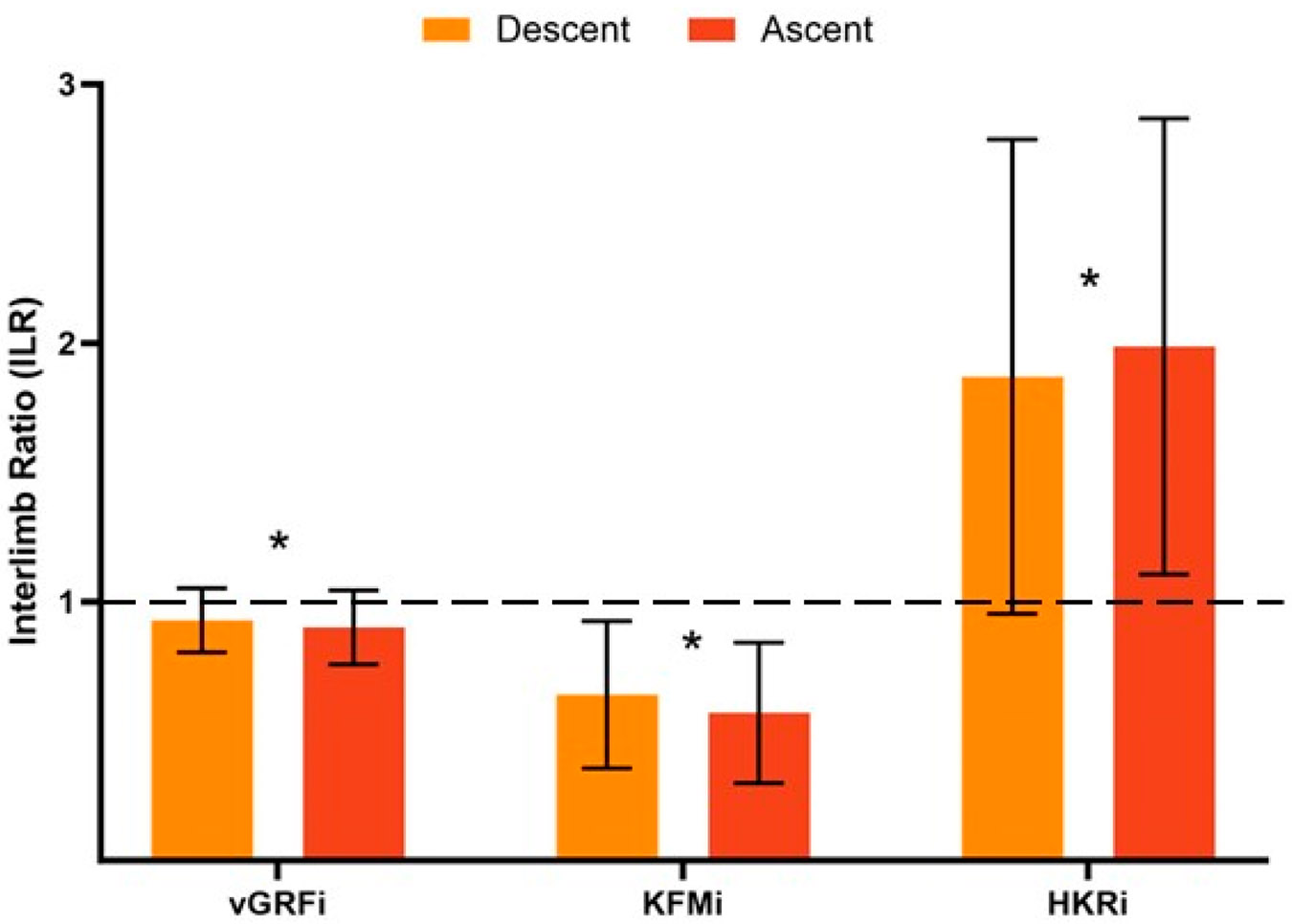
Interlimb ratios for vertical ground reaction force impulse, external knee flexion moment impulse and ratio of external hip flexion to knee flexion moment impulse during the descent and ascent phases of squats. Error bars represent ±1 standard deviation. * *p*-value of <0.05. Note: The dashed line represents an interlimb ratio of 1 and an equal distribution of loading for each variable between the injured and uninjured limb. Abbreviations: ILR, interlimb ratio; vGRFi, vertical ground reaction force impulse; KFMi, external knee flexion moment impulse; HKRi, ratio of external hip flexion to knee flexion moment impulse.

Forty participants were initially enrolled in the study. Five participants were lost to follow-up by 6 months after ACLR, resulting in thirty-five participants included in this analysis. Descriptive statistics for sex, age, race, weight, height and BMI of the included participants are presented in Table 1. More than half of the participants (54%) had a concomitant meniscal repair. Nearly two-thirds (63%) of participants had a patellar tendon autograft during ACLR. Overall limb loading (vGRFi) was more symmetric compared to knee-specific loading contributions (KFMi, HKRi) (Table 2, Figure 2).

There was a significant difference in loading asymmetry between the descent and ascent phases of bilateral squats for all biomechanical variables (Table 3). Participants demonstrated more asymmetry during the ascent phase for vGRFi ILR (*p* = 0.045; Descent (D): 0.93 ± 0.12, Ascent (A): 0.90 ± 0.14), KFMi ILR (*p* < 0.005; D: 0.70 ± 0.39, A: 0.64 ± 0.39) and HKRi ILR (*p* = 0.006; D: 1.84 ± 0.93, A: 2.06 ± 1.12) (Figure 1). There was a significant difference in time with the ascent phase being faster than the descent phase (*p* = 0.036; D:1.00 ± 0.25 s, A:0.96 ± 0.19 s) (Figure 2).

After adjusting for time during each phase, there was no longer a statistically significant difference in vGRFi ILR (*F*(1, 67) = 0.581, *p* = 0.449, partial η^2^ = 0.009), KFMi ILR (*F*(1, 67) = 0.579, *p* = 0.449, partial η^2^ = 0.009) or HKRi ILR (*F*(1, 61) = 0.733, *p = 0*.395, partial η^2^ = 0.012). Adjusted means and variability for each of the biomechanical variables are provided in Table 3.

## 4. Discussion

Our results indicate that at 6 months after ACLR, participants continue to exhibit deficits in knee loading symmetry during both the descent and ascent phases of squatting. Initially, greater asymmetry was observed during the ascent phase, as evidenced by vGRFi, KFMi and HKRi, compared to the descent phase. Specifically, the injured limb demonstrated approximately 30% lower external knee flexion moment impulse compared to the uninjured limb, along with nearly twice the hip flexion moment contribution relative to knee flexion moment contribution (HKRi). However, given that the ascent phase was performed at a faster speed than the descent (4% faster), we controlled for movement time to account for potential confounding. After adjusting for time, interlimb loading asymmetries persisted, but the previously observed difference in asymmetry between the ascent and descent phases was no longer statistically significant.

The findings of our study align with and expand upon previous research. Sigward et al., Sanford et al. and Garrison et al. reported asymmetries when analyzing the squat as a single movement, while Strong et al. identified phase-specific asymmetries when comparing the limb of participants after ACLR to a healthy control limb [1,4,5,9]. In our study, asymmetries were evident in both phases; however, they were more pronounced during the ascent phase, which may be attributed to several factors. First, participants were instructed to perform squat sets without standardized control over time under tension and phase-specific velocity. Our findings indicate that differences in movement velocity across phases may influence an individual’s control over the squatting movement, particularly during the ascent phase, thereby highlighting pre-existing asymmetrical movement patterns. Additionally, neuromuscular control of the lower limb and muscle activation are critical factors in squat execution during rehabilitation after ACLR. It is well established that neural control patterns differ across eccentric compared to concentric muscle contractions, with a lower rate of motor unit recruitment required under equal force conditions during eccentric versus concentric muscle contraction [21]. ACL injury and subsequent ACLR often result in arthrogenic muscle inhibition (AMI), a neural impairment that restricts proper quadriceps activation in the affected limb that is still present up to 2 years after ACLR [22,23]. Given that greater quadriceps activation is required during the ascent phase compared to the descent phase of a bodyweight squat, reduced quadriceps neuromuscular activation may contribute to the greater asymmetry observed in this phase [24]. Finally, the depth of the squats was selected at the discretion of the participants according to their comfort and ability. Deeper squats have been associated with increased muscle activation, particularly in the quadriceps and gluteus maximus [25]. Therefore, variations in squat depth, along with potential improper muscle activation, may contribute to greater asymmetries during the ascent phase compared to the descent phase.

### Strengths and Limitations

This is the first study to compare loading asymmetries between the injured and uninjured limbs during the descent and ascent phases of bilateral squats after ACLR. By breaking down the movement into its directional components, we gain novel insight into the specific biomechanical strategies patients use after ACLR, which could inform more targeted rehabilitation protocols to address persistent deficits. Limitations of our study include a relatively small sample size of athletic participants with a young average age (19 years). Therefore, our findings may not be generalized to older populations. In addition, the differences in vGRFi, KFMi and HKRi interlimb ratios between the descent phase and ascent phase were relatively small; it is unknown if these differences are clinically meaningful. Finally, not controlling squat depth across participants aided in the generalizability of our study findings to real-world movements but could have impacted findings.

## 5. Conclusions

At 6 months after ACLR, interlimb asymmetries persist during both the descent and ascent phases of the squat, with greater asymmetries observed during the ascent phase. However, these asymmetries were no longer present after controlling for phase duration. This suggests that underlying neuromuscular control deficits remain even during controlled bodyweight activities at this stage of the rehabilitation process. Furthermore, movement speed during rehabilitation exercises should be carefully considered to effectively mitigate biomechanical imbalances in closed kinetic chain movements.

## Figures and Tables

**Table 1.** Participant and surgical characteristics.

	Number of Participants (%) or Mean (SD)	95% Confidence Interval
Sex (female)	21 (60.0)	
Age (years)	19.7 (4.6)	18.1–21.3
Race		
Asian	2 (5.7)	
Black or African American	3 (8.6)	
Hispanic, Latino or Spanish	3 (8.6)	
White	25 (71.4)	
Other	2 (5.7)	
Weight (kg)	74.2 (16.3)	68.6–79.8
Height (m)	1.7 (0.1)	1.7–1.7
Body mass index (BMI; kg/m^2^)	25.9 (4.8)	24.2–27.5
Concomitant meniscus repair (Yes)	19 (54.3)	
Graft type		
Hamstring tendon autograft	4 (11.4)	
Patellar tendon autograft	22 (62.9)	
Quadriceps tendon autograft	8 (22.9)	
Allograft	1 (2.9)	

Abbreviations: %, percentage; SD, standard deviation; kg, kilograms; m, meters; BMI, body mass index.

**Table 2.** Results of interlimb ratios for vertical ground reaction force impulse, external knee flexion moment impulse and ratio of external hip flexion to knee flexion moment impulse, as well as time during the different phases of squats.

	Descent (Mean ± SD)			Ascent (Mean ± SD)			*p*-Value
	Injured	Uninjured	ILR	Injured	Uninjured	ILR	
vGRFi (BW/kg·m)	0.48 ± 0.12	0.52 ± 0.13	0.93 ± 0.12	0.45 ± 0.10	0.51 ± 0.10	0.90 ± 0.14	0.045 *
KFMi (N·m·s/kg·m)	0.22 ± 0.10	0.35 ± 0.15	0.70 ± 0.39	0.18 ± 0.09	0.32 ± 0.11	0.64 ± 0.39	<0.001 *
HKRi (N·m·s/kg·m)	1.74 ± 0.71	1.12 ± 0.53	1.84 ± 0.93	2.04 ± 0.84	1.17 ± 0.53	2.06 ± 1.12	0.006
Time (s)		1.00 ± 0.25			0.96 ± 019		0.036 *

* *p*-value of <0.05. Abbreviations: SD, standard deviation; ILR, interlimb ratio; vGRFi, vertical ground reaction force impulse; BW, bodyweight; s, seconds; kg, kilograms; m, meters; KFMi, external knee flexion moment impulse; N, newtons; HKRi, ratio of external hip flexion to knee flexion moment impulse.

**Table 3.** Results of paired t-tests comparing the vertical ground reaction force impulse, external knee flexion moment impulse, ratio of external hip flexion to knee flexion moment impulse and time in each squat phase during the descent compared to ascent phase of bilateral squats.

		Mean Difference	SD	SEM	95% CI	*p*
vGRFi ILR	Unadjusted	0.029	0.083	0.014	0.001 to 0.058	0.045 *
	Adjusted	0.024	-	0.032	−0.039 to 0.088	0.449
KFMi ILR	Unadjusted	0.066	0.095	0.016	0.034 to 0.099	<0.005 *
	Adjusted	0.071	-	0.093	−0.115 to 0.257	0.449
HKRi ILR ^†^	Unadjusted	−0.218	0.418	0.074	−0.368 to −0.067	0.006 *
	Adjusted	−0.223	-	0.260	−0.744 to 0.298	0.395
Time (s)	-	0.047	0.129	0.022	0.003 to 0.092	0.036 *

* *p*-value of <0.05.

† Three extreme outliers were not included in this analysis (*n* = 32). Abbreviations: SD, standard deviation; SEM, standard error of the mean; s, seconds; t, t-value; df, degrees of freedom; vGRFi, vertical ground reaction force impulse: KFMi, external knee flexion moment impulse; HKRi, ratio of external hip flexion to knee flexion moment impuse; ILR, interlimb ratio.

## Data Availability

The data that support the findings of this study are available from the corresponding author upon reasonable request. The data are not publicly available due to privacy.
